# Resveratrol attenuates inflammation and fibrosis in rheumatoid arthritis-associated interstitial lung disease via the AKT/TMEM175 pathway

**DOI:** 10.1186/s12967-024-05228-1

**Published:** 2024-05-14

**Authors:** Nannan Liu, Xuefei Fan, Yubao Shao, Suhuan Chen, Taorong Wang, Tao Yao, Xiaoyu Chen

**Affiliations:** 1 Department of Histology and Embryology, Anhui Medical University, No. 81 Meishan Road, Hefei, 230032 Anhui China; 2 Department of Orthopedics, The Third Affiliated Hospital of Anhui Medical University, No. 390 Huaihe Road, Hefei, 230061 Anhui China

**Keywords:** RA-ILD, Resveratrol, Autophagy, TMEM175

## Abstract

**Background and purpose:**

Interstitial lung disease (ILD) represents a significant complication of rheumatoid arthritis (RA) that lacks effective treatment options. This study aimed to investigate the intrinsic mechanism by which resveratrol attenuates rheumatoid arthritis complicated with interstitial lung disease through the AKT/TMEM175 pathway.

**Methods:**

We established an arthritis model by combining chicken type II collagen and complete Freund’s adjuvant. Resveratrol treatment was administered via tube feeding for 10 days. Pathological changes in both the joints and lungs were evaluated using HE and Masson staining techniques. Protein expression of TGF-β1, AKT, and TMEM175 was examined in lung tissue. MRC-5 cells were stimulated using IL-1β in combination with TGF-β1 as an in vitro model of RA-ILD, and agonists of AKT, metabolic inhibitors, and SiRNA of TMEM175 were used to explore the regulation and mechanism of action of resveratrol RA-ILD.

**Results:**

Resveratrol mitigates fibrosis in rheumatoid arthritis-associated interstitial lung disease and reduces oxidative stress and inflammation in RA-ILD. Furthermore, resveratrol restored cellular autophagy. When combined with the in vitro model, it was further demonstrated that resveratrol could suppress TGF-β1 expression, and reduce AKT metamorphic activation, consequently inhibiting the opening of AKT/MEM175 ion channels. This, in turn, lowers lysosomal pH and enhances the fusion of autophagosomes with lysosomes, ultimately ameliorating the progression of RA-ILD.

**Conclusion:**

In this study, we demonstrated that resveratrol restores autophagic flux through the AKT/MEM175 pathway to attenuate inflammation as well as fibrosis in RA-ILD by combining in vivo and in vitro experiments. It further provides a theoretical basis for the selection of therapeutic targets for RA-ILD.

**Graphical Abstract:**

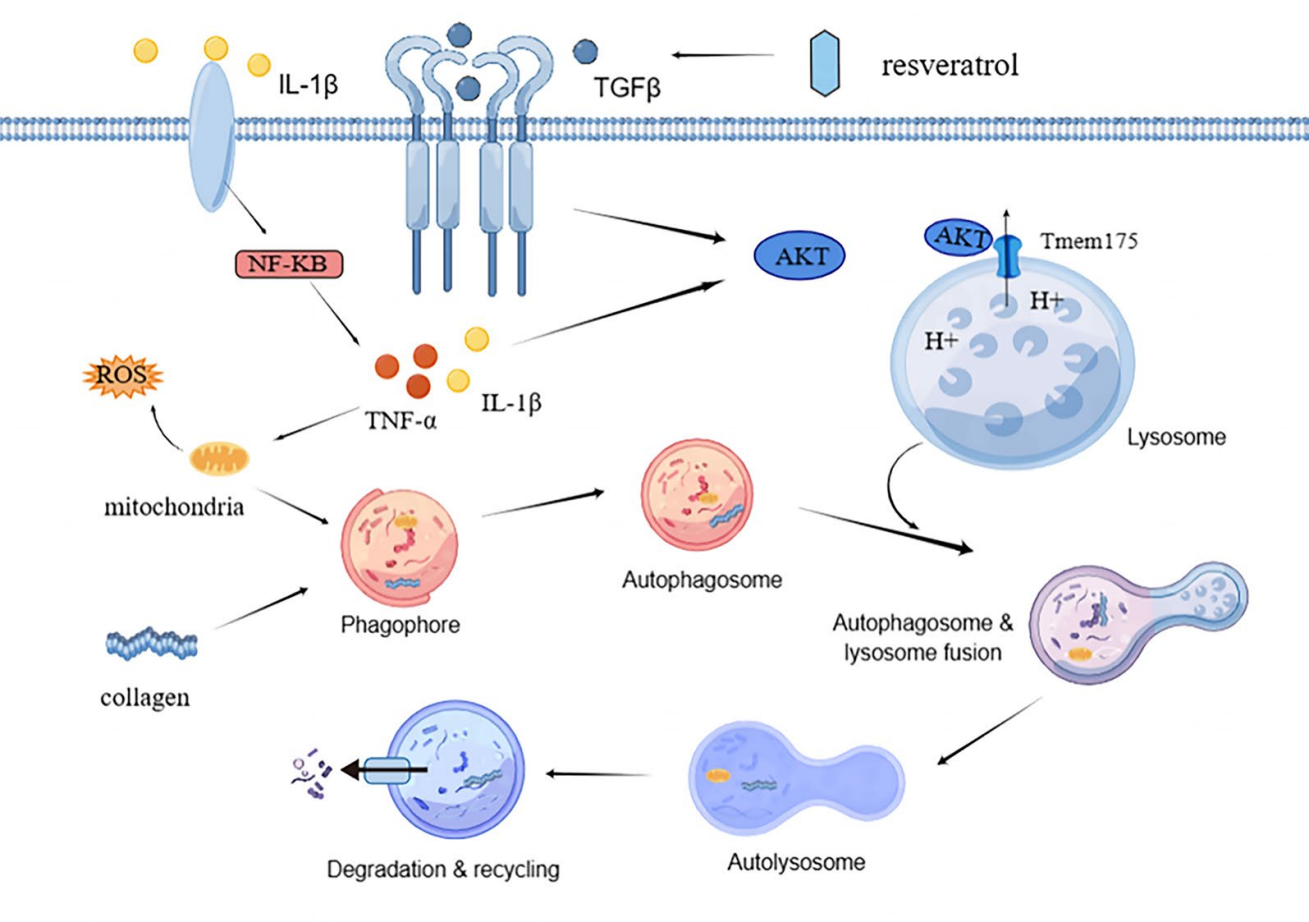

## Introduction

Rheumatoid arthritis (RA) is an autoimmune disease with an unknown etiology. Its primary characteristics include chronic inflammation and hyperplasia of the synovial membrane within joints, which in turn affects the articular cartilage, resulting in cartilage damage, and destruction, and ultimately leading to joint deformities and functional loss [1, 2]. Rheumatoid arthritis extends beyond the joints; the lungs, with their abundant connective tissue and blood supply, are commonly affected organs. In recent years, there has been a growing focus on the investigation of interstitial lung disease [3, 4]. Rheumatoid arthritis interstitial lung disease is frequently undetected during its early stages due to the lack of apparent clinical symptoms. Furthermore, the prognosis for pulmonary fibrosis in advanced stages is exceedingly grim [5]. The primary pathological manifestations of RA-ILD include localized fibroblast proliferation, accumulation of extracellular matrix, and the development of alveolar cystic-cavity-like dilatation in lung tissue. These changes result in the distortion of the typical lung structure and a decrease in the effective air exchange area in the distal region. Consequently, the majority of patients eventually succumb to hypoxic respiratory failure [6].

Resveratrol is a naturally occurring polyphenol, abundant in grapes, known for its potent antioxidant, anti-inflammatory, and antibacterial properties [7]. In a particular study, it was discovered that resveratrol decreased synovial proliferation, lowered inflammatory markers, and mitigated oxidative damage in an experimentally induced arthritis model [8, 9]. Resveratrol can suppress inflammatory responses, and cellular proliferation, and induce synovial cell apoptosis by inhibiting the MAPK signaling pathway and diminishing ROS accumulation [10]. Additionally, resveratrol enhances rheumatoid arthritis by triggering the SIRT1-Nrf2 signaling pathway [11]. While the mechanism of resveratrol in mitigating rheumatoid arthritis has been extensively documented, its potential to attenuate the progression of RA-ILD has received relatively less attention. In this study, we investigated the potential of resveratrol as a therapeutic agent for RA-ILD.

Autophagy is a cellular process that involves the degradation of dysfunctional cellular components through the action of lysosomes within the cell [12]. Autophagy is a cellular process that involves the degradation of dysfunctional cellular components through the action of lysosomes within the cell [13, 14]. Recently, the role of autophagy in autoimmune diseases has garnered significant attention and recognition [15]. Notably, research in the context of rheumatoid arthritis has revealed, for instance, that autophagy triggers protein aminoacylation in fibroblast-like synovial cells derived from individuals with rheumatoid arthritis [16]. Autophagy mitigates joint damage caused by synovial cells through the involvement of optineurin [17]. Furthermore, autophagy plays a significant role in the development of pulmonary fibrosis [18–20]. Research has demonstrated that resveratrol can suppress fibrosis in RA-ILD by reinstating autophagy [21].

TMEM175 is a K^+^ channel protein primarily found within nuclear endosomes and lysosomes. Its main function is facilitating K^+^ transmembrane transport [22]. Nevertheless, it has been discovered that TMEM175 possesses not only a K^+^ transporter function but also a notably robust H^+^ transporter function [23, 24]. Protein kinase B (Akt), alternatively referred to as PKB or Rac, holds a pivotal role in regulating cell survival and apoptosis [25]. Growth and survival factors, including insulin, can activate the Akt signaling pathway [26]. Research revealed that AKT associates with TMEM175 to create a lysosomal K-channel complex. This complex is activated by growth factors and plays a role in regulating lysosomal K^+^ and H^+^ transport through AKT gating, consequently controlling lysosomal pH [27–29].

The objective of this study was to explore the function and underlying mechanism of resveratrol in alleviating rheumatoid arthritis in the presence of interstitial lung disease. Specifically, this mechanism involves the restoration of autophagic lysosomal flux through the AKT/TMEM175 pathway.

## Materials and methods

### Animal model

Eight-week-old male C57BL/6 mice with a body weight ranging from 20 to 25 g were randomly assigned to one of three groups: (I) Ctrl (Control), (II) CIA (collagen-induced arthritis models), and (III) CIA + Res (CIA models treated with resveratrol at 10 mg/kg). Male C57BL/6 mice were procured from Jiangsu Ji Cui Biotechnology Co., Ltd. (Nanjing, China) and acclimated to laboratory conditions at Anhui Medical University for a minimum of 1 week before the experiment. The induction of CIA in mice involved the use of complete Freund’s adjuvant (CFA) and chicken type II collagen (CCII). CCII (Chondrex, Woodinville, WA, USA) at a concentration of 2 g/L was dissolved in 0.05 mol/L glacial acetic acid, thoroughly stirred, and stored at 4 °C overnight. Subsequently, CFA (Chondrex, Woodinville, WA, USA) was mixed with an equivalent volume of the dissolved Col II and completely emulsified in an ice bath using a homogenizer. For the CIA + Res group, animals received resveratrol (Aladdin, Shanghai, China) via oral gavage, dissolved in 0.5% sodium carboxymethylcellulose (carrier) at a final concentration of 1 mg/mL, with a daily dosage of 0.1 mL per 10 g of body weight, administered for 10 days. The control group received an equivalent volume of normal saline via tracheal and tail root injection, serving as the negative control. The resveratrol dosage was determined based on prior research findings [30]. All animals were euthanized 24 h after the final resveratrol treatment. Lung tissue was excised, with a section reserved for pathological analysis. The remaining lung tissue was promptly frozen in liquid nitrogen to facilitate subsequent bioanalysis.

### Hematoxylin–Eosin (H&E) staining

Following fixation with 4% neutral paraformaldehyde, knee tissue specimens underwent a 2-week immersion in EDTA decalcification solution (Boster, Wuhan, China) under room temperature agitation. Subsequently, they were subjected to standard dehydration and paraffin embedding procedures. Paraffin sections were approximately 3.5 µm thick, and these sections were deparaffinized and subjected to a series of alcohol and distilled water washes. Hematoxylin staining was performed for 15 s, followed by a 20-min tap water rinse to counterstain. Subsequently, 5% eosin staining for 3 min was carried out, and the sections were rapidly dehydrated. Finally, a neutral resin was used to seal the slides. Lung tissue specimens followed a similar process, including fixation, dehydration, paraffin embedding, and sectioning at a thickness of approximately 3.5 µm. HE staining was performed on these lung tissue sections as well.

### Masson staining

Masson staining (Solarbio, Beijing, China) was employed to identify fibrosis. The cells were subjected to iron hematoxylin staining for 8 min at room temperature for nuclear staining, followed by differentiation to achieve a blue color. Subsequently, they were stained with aniline blue for 1.5 min, washed with weak acid, dehydrated, and sealed. Finally, observations were made under a microscope (Nikon Eclipse 80I), and analysis was conducted using Image Pro 6 (Media Contronetics Inc., Bethesda, MD, USA).

### Immunohistochemistry

Following the standard dewaxing of paraffin sections, endogenous peroxidase activity was quenched by incubation with 3% H_2_O_2_ for 20 min. Subsequently, antigen retrieval was performed using a microwave for 10 min, and the primary antibody was incubated at 4 °C overnight. The addition of the secondary antibody was followed by a 60-min incubation at room temperature. DAB (Solarbio, Beijing, China) was utilized for color development, and the slides were counterstained with hematoxylin before sealing with neutral resin. Five randomly selected areas within each section were examined under a light microscope. Positive expression was indicated by yellow–brown staining in the cytoplasm or cytoplasmic membrane. The proportion of positive area (positive area/total area) was quantified using ImageJ software.

### Hydroxyproline assay

Following the kit instructions (Solarbio, Beijing, China), approximately 0.2 g of the sample was weighed into a glass tube. The tissue was minced as finely as possible for digestion, and 2 mL of extraction solution was added before bringing it to a boil. After cooling, the pH was adjusted to the range of 6–8 using 10 mol/L NaOH (approximately 1 mL), and then distilled water was added to reach a final volume of 4 mL. Subsequently, centrifugation was carried out at 16,000 rpm and 25 °C for 20 min. The spectrophotometer wavelength was set to 560 nm, and the supernatant was collected for measurement.

### Malondialdehyde assay

Approximately 0.1 g of tissue was weighed and homogenized in 1 mL of extraction solution on ice. After centrifugation at 8000×*g* for 10 min at 4 °C, the supernatant was collected and kept on ice for subsequent measurements. Following the kit instructions (Solarbio, Beijing, China), 200 μL of the supernatant was pipetted into either a micro glass cuvette or a 96-well plate, and the absorbance of each sample was measured at 532 nm and 600 nm.

### Molecular docking

Three-dimensional structural representations of the crucial active components of resveratrol were acquired from the PubChem database to serve as ligand files for subsequent molecular docking. The 3D structures of the target proteins were retrieved as receptor files from the RCSB database (https://www.pdbus.org/). AutoDock (version 1.5.6) was employed to extract small ligand molecules from the receptors, perform side chain corrections, and introduce hydrogen atoms. Subsequently, AutoDock Vina (version 1.1.2) was utilized for molecular docking. A protein binding affinity below − 5 kcal/mol indicates significant binding activity with the compound. The docking outcomes for the compounds and proteins displaying the most favorable conformations were subjected to analysis and visualization using PyMol software.

### Molecular dynamics simulations of target protein complexes

Gromacs was utilized to conduct molecular dynamics simulations on the resveratrol-TGF-β1 protein complex, which exhibited the highest binding free energy in molecular docking. Protein modeling employed aber14SB force field parameters, while small molecule ligands were modeled with Gaff generic force field parameters. The OPC water model was employed to introduce solvents into the protein–ligand system. Subsequently, a water tank was established and supplemented with Na^+^ and Cl^−^ to achieve system equilibrium. Energy optimization was carried out employing the most rapid descent and conjugate gradient methods. Conformational constraints were applied, followed by Nvt pre-equilibrium and Npt pre-equilibrium, culminating in MD simulations. Binding free energy serves as the primary criterion for assessing drug molecule activity, with lower values indicating greater complex stability.

### Cell lines and cell culture

The human embryonic lung fibroblast cell line (MRC-5) was procured from the American Typical Collection of Biological Resources (ATCC, Rockville, MD, USA) and cultured in Dulbecco’s modified Eagle’s medium (DMEM, Life Technologies/Gibco, Grand Island, NY, USA) supplemented with 10% fetal bovine serum (Gibco) and 1% antibiotics (containing 100 U/mL penicillin, 100 μg/mL streptomycin, Sigma-Aldrich, St. Louis, MO, USA). The cells were maintained in a 37 °C incubator with 5% CO_2_. To induce a cellular model of RA-ILD, recombinant TGF-β1 and IL-1β were added to the cell culture medium for a 24-h incubation period. TGF-β1, IL-1β, SC79, MK2206, chloroquine (CQ), and bafilomycin A1 (BAFA1) were procured from MedChemExpress, while MG132 was obtained from Selleckchem.

### Cell transfection and processing

SiRNA targeting TMEM175 (Si-TMEM175) was custom-designed and synthesized by General Biologicals. Cells were transfected with SiRNA at a concentration of 50 nmol/L using Lipo2000 (Thermo Fisher Scientific, Waltham, MA, USA) following the manufacturer’s guidelines. Subsequently, the transfected cells were utilized for experiments. The sequence of Si-TMEM175 is provided in Table 1.

**Table 1:** Small interfering RNA and primer sequences

Name	Sequences
Si-NC	Sense UUCUCCGAACGUGUCACGUTT
	Antisense ACGUGACACGUUCGGAGAATT
Si-TMEM175	Sense GCAGGUUCAGUGUGGGCAUTT
	Antisense AUGCCCACACUGAACCUGCTT

	Primer sequence
PKC-A	Forward CTGTTCCCACCCTATCACTCC
	Reverse GCTCGGAAGCCTCATAAGAT
PKC-B	Forward ACCAAAAGCTAGAGACAAGCGA
	Reverse GTGGGAGTCAGTTCCACAGG
PKC-D	Forward GCCTTCCATGGCTTCTCCTT
	Reverse CCAGTCACCCACTGTTCTCC
GSK3B	Forward CAGGACATTTCACCCCAGGA
	Reverse AGGTGTGTCTCGCCCATTTG
TFEB	Forward CGCAGAGAACGGAGACGG
	Reverse TGAAGGTCAATCTGTCCGCC
RAB7A	Forward CGTGACAGACACTTCCGCT
	Reverse AACGGAGGAGGAGACACAAC
TBC1D15	Forward GAAAGCTGTTGTGGGCCATT
	Reverse CTGCCTCGCAAACTGTCAAA
FIS1	Forward CAGTTGCGTGTGTTAAGGGATGAA
	Reverse TTCAAAATTCCTTGCAGCTTCGT
VAMP8	Forward TGCCTTGGGTGGAAACAGAC
	Reverse TGCAGGTTCCTAACTCGGTC
SNAP29	Forward TAGATGAGCTGTCCGTGGGA
	Reverse TGGTTGTCAGTCGGTCAAGG
STX17	Forward CTAGGCGGGAGGTGTTTCTG
	Reverse AGCCTGCGTAACTTCACCTT
VATPASE	Forward CTGGTTCGAGGATGCAAAGC
	Reverse TCCAAGGTCTCACACTGCAC
ATP6AP1	Forward CAGGACAAGAATGCCCTGGAC
	Reverse CTGTGTAGGGCAGACGGATG
ATP6AP2	Forward GGACCATTCACCCGACTTGT
	Reverse CACTGCGTTCCCACCATAGA
TMEM175	Forward CGATCCTACGGACCTCAAGC
	Reverse GACCAACAAGTTGTCACGGC
TNF-α	Forward ACCCTCACACTCACAAACCA
	Reverse ACAAGGTACAACCCATCGGC
IL-1β	Forward TGCCACCTTTTGACAGTGATG
	Reverse AAGGTCCACGGGAAAGACAC
β-Actin	Forward CAGCCTTCCTTCTTGGGTATGG
	Reverse CGCAGCTCAGTAACAGTCCG

### Cell counting kit-8 (CCK-8) assay

MRC-5 cells were seeded in 96-well plates at a density of 3 × 10^3^ cells per well. After 6–12 h, the cells were stimulated with IL-1β and TGF-β1 for 24 h. Subsequently, following a 24-h treatment with resveratrol, the cells were further incubated for 1–4 h with the addition of 10 μL of CCK-8 solution (Biosharp, Beijing, China) to each well. The absorbance of the cells at 450 nm was assessed using an enzyme-linked marker to evaluate the impact of resveratrol on cell viability.

### EDU cell proliferation assay

Culture an appropriate cell number in a 6-well plate. Once the cell culture has returned to its normal state overnight, proceed with the desired treatments. Prepare the EdU working solution (Beyotime Biotechnology, Shanghai, China) and add the preheated EdU working solution at 37 °C to the 6-well plate, incubating the cells for 2 h. Fix the Edu-labeled cells by adding 1 mL of fixative and incubating for 15 min at room temperature. Subsequently, incubate each well with 1 mL of permeabilizing solution for 10–15 min at room temperature. Add 0.5 mL of Click reaction solution to each well and incubate for 30 min at room temperature, away from light. Stain the cell nuclei with Hoechst 33342, followed by fluorescence detection.

### Detection of cell ROS

ROS levels were assessed using the DCFH-DA green fluorescent probe (Beyotime Biotechnology, Shanghai, China). DCFH-DA was diluted 1000-fold with DMEM to create a working solution. Subsequently, 1–2 mL of the working solution was added to completely cover the cells growing on the culture dish and incubated at 37 °C for 20 min in the absence of light. Afterward, the cells were washed three times with PBS buffer and imaged using an inverted fluorescence microscope. The alterations in ROS levels in MRC-5 cells were analyzed using ImageJ software.

### Mitochondrial membrane potential level detection

The mitochondrial membrane potential was assessed using the mitochondrial membrane potential kit from Beyotime Biotechnology (Shanghai, China). Cells were seeded and incubated overnight at 37 °C. Following the instructions provided with the JC-1 reagent kit, JC-1 was diluted 1000-fold to prepare the working solution. Subsequently, 1–2 mL of the working solution was added to fully cover the cells on the culture dish and incubated at 37 °C in the absence of light for 15 min. The cells were then washed three times with PBS buffer. Images were captured using an inverted fluorescence microscope, and changes in the membrane potential of MRC-5 cells were analyzed using ImageJ software.

### mCherry-GFP-LC3 dual fluorescence tracks autophagosome alterations

Cells were transiently transfected with the mCherry-GFP-LC3 construct using EZ Cell Transfection Reagent (Shanghai Life-iLab Biotech, AC04L091). Transfection was performed by adding 3 μg of mCherry-GFP-LC3 plasmid to 9 μL of the transfection reagent, and the culture medium was refreshed every 24 h. After 48 h of cell model treatment, the number of mCherry and GFP fluorescent puncta was directly observed using a laser confocal microscope (Leica SP8, Wetzlar, Germany).

### MDC staining

The cells to be examined were subjected to three washes with PBS. Subsequently, 1 mL of MDC staining solution (Beyotime Biotechnology, Shanghai, China) was added to each well, and the cells were incubated in a cell culture incubator at 37 °C for 30 min while protected from light. Afterward, the cells were washed three times with Assay Buffer, with each wash involving 0.8–1 mL of Assay Buffer. Finally, the Assay Buffer was aspirated, and 1 mL of fresh Assay Buffer was added. The green fluorescence was observed using a fluorescence microscope with UV excitation light.

### Lyso-tracker red staining

A small quantity of Lyso-Tracker Red (Beyotime Biotechnology, Shanghai, China) was added to the cell culture medium at a ratio of 1:20,000, resulting in a final concentration of 50 nM. The cell culture medium was then aspirated, and the prepared working solution was added, followed by incubation at 37 °C for 30 min. Subsequently, the Lyso-Tracker Red stained working solution was removed, and a fresh cell culture medium was added. Photographic observation was carried out using a laser confocal microscope.

### Western blotting

Total protein was extracted from mouse lung tissues and cells using a prepared SDS lysis buffer for protein blotting analysis. The proteins were separated on SDS–polyacrylamide gels and transferred to a polyvinylidene fluoride membrane (PVDF, Merck Millipore, Germany). The membrane was then blocked with 5% skimmed milk for 2 h. Antibodies against β-actin (Abcam, #ab8226), Collagen I (Arigo Biolaboratories, #ARG21965), TGF-β1 (ImmunoWay, #YT4632), P-NFKB (Wanleibio, #WL02169), NFKB (Bioss, #bsm-33117M), TNF-α (Proteintech, #60291-1-Ig), IL-1β (Wanleibio, #WLH3903), HIF-1a (Proteintech, #20960-1-AP), SOD1 (Wanleibio, #WL01846), SOD2 (Boster, #BA4566), LC3-II (Cell Signaling Technology, #12741), P62 (Cell Signaling Technology, #16177S), LAMP2 (Proteintech, #66301-1-Ig), P-mTOR (Abmart, #T56571), mTOR (Abmart, #T55306), P-ULK1 (ImmunoWay, #YP1544), ULK1 (Abmart, #T56902), Beclin1 (Boster, #PB0014), Atg5 (Bioss, #bs-4005r), BNIP3 (Santa Cruz Biotechnology, #sc-56167), P-AKT (Abmart, #T40067), AKT (Abmart, #BM4400), and TMEM175 (Proteintech, #19925-1-AP) were used. The PVDF membrane was incubated with these antibodies at 4 °C overnight. Subsequently, an HRP-labeled secondary antibody (ZSGB-BIO, Beijing, China) was added and incubated for 1 h, followed by detection using the ECL luminescence system.

### Co-inmunoprecipitation assay

Transfer 25–50 μL of protein A/G magnetic beads (GenScrip, New Jersey, USA) into 1.5 mL tubes and add 400 μL of Wash Buffer (PBST) to the beads. Dilute Antibody TMEM175 with Wash Buffer to achieve a final concentration of 5–50 μg/mL. Combine 400 μL of the diluted Ab with the Protein A/G beads and incubate at 2 °C for 4 h with rotation. Wash the beads by adding 400 μL of Wash Buffer and gently agitating. Place the tube in a magnetic holder, collect the beads on the side of the tube, discard the supernatant, and repeat this step 4 times. Remove the tube from the magnetic separator, add the sample containing the antigen (Ag), and gently pipette to resuspend the protein A/G bead-Ab complex. Incubate at 2 °C for 4 h to allow Ag to bind to the Protein A/G Bead-Ab complex. Add 400 μL of Wash Buffer to the tube, spin for 5 min, perform a 1-min magnetic separation, and remove the supernatant. Finally, add 25–50 μL of elution buffer to the tube containing the magnetic bead-Ab-Ag complex and spin for 5 min to elute the protein complex.

### Immunofluorescence assay

MRC-5 cells were plated in 48-well plates at a density of 1 × 10^4^ cells per well. After 48 h of cell treatment, the cells were fixed with 4% paraformaldehyde for 30 min at room temperature and then rinsed with PBS. Subsequently, the cells were treated with 0.1% Triton-X for 20 min and rinsed again. Afterward, they were blocked with 5% bovine serum albumin (BSA, Solarbio, Beijing, China) for 60 min at room temperature, followed by incubation with antibodies (diluted 1:200) such as LC3-II, P62, etc., overnight at 4 °C. The cells were then rinsed and incubated for 1 h in the dark with secondary antibodies (goat anti-mouse antibody, Proteintech or goat anti-rabbit antibody, Proteintech). Subsequently, DAPI (Beyotime Biotechnology, Shanghai, China) was added to the cells for a 10-min incubation in the dark. Finally, the samples were sealed with an anti-fading mounting medium (Beyotime Biotechnology, Shanghai, China) and observed using an inverted fluorescence microscope.

### Total RNA isolation and quantitative real-time reverse transcription PCR (qRT-PCR)

Total RNA was isolated from mouse lung tissue using TRIzol reagent (Thermo Fisher Scientific, Waltham, MA, USA). TRIzol was added to the lung tissue powder and allowed to stand at room temperature for 5 min. Subsequently, 200 μL of chloroform was added to each sample and vortexed. After a 5-min incubation, the samples were centrifuged at 12,000 rpm and 4 °C for 15 min. The upper aqueous phase was transferred, and 400 μL of isopropanol was added, followed by a 10-min incubation and centrifugation at 12,000 rpm and 4 °C for 10 min to collect the RNA pellet. The RNA pellet was washed with 70% ethanol, centrifuged, air-dried, and then resuspended in DEPC water. Finally, RNA concentration and purity were determined using UV spectrophotometry. Reverse transcription of total RNA was performed according to the instructions of the cDNA Reverse Transcription Kit. Quantitative PCR was carried out using SYBR Green (Beyotime Biotechnology, Shanghai, China) on a Roche LightCycler 480 real-time fluorescent quantitative PCR system. After completion of the reaction, the cycle threshold (Ct) values were determined, and the relative RNA levels of each sample were calculated using the 2^−ΔΔCt^ method, with normalization to β-actin levels. Primer sequences are provided in Table 1.

### Flow cytometric analysis

Cell culture and treatment followed the same protocol as described above. Single-cell suspensions were prepared and fixed in 70% ethanol by volume. RNase A was added, followed by a 30-min incubation in a 37 °C water bath. Then, PI (Bestbio, Shanghai, China) staining was added, and mixed, and the samples were kept in the dark at 4 °C for 30 min. Flow cytometry (BD Celesta, USA) was conducted to record red fluorescence with an excitation wavelength of 488 nm.

### Statistical analysis

Continuous variables are presented as mean ± standard deviation (SD). Overall significance was assessed using one-way ANOVA with GraphPad Prism 5 (GraphPad Software, La Jolla, CA, USA). A significance level of *p* < 0.05 was considered statistically significant when comparing differences between groups.

## Results

### Resveratrol alleviates RA-ILD fibrosis

**Fig. 1:**
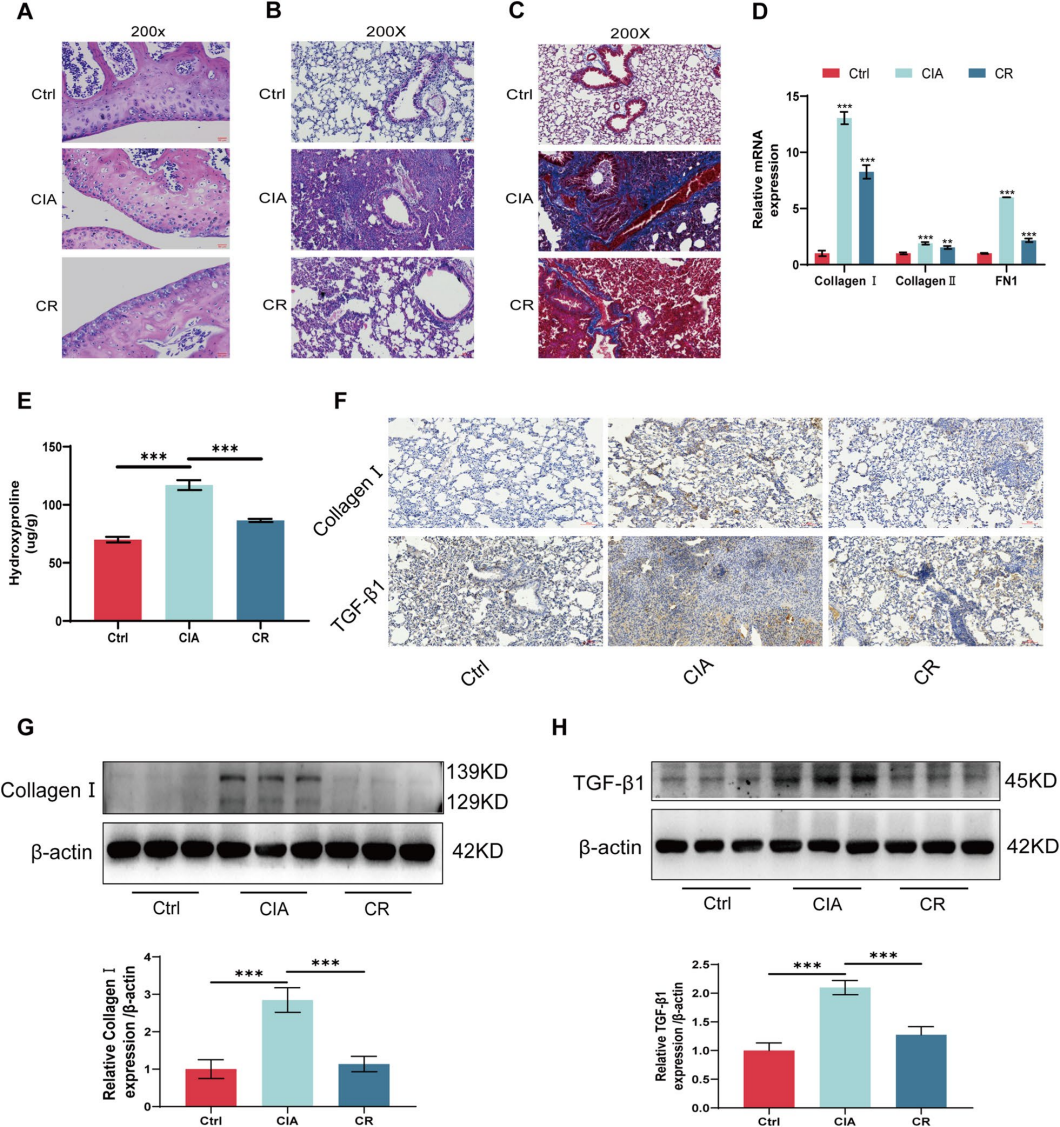
Resveratrol alleviates RA-ILD fibrosis. **A** 8-week-old male C57BL/6 mice were randomly divided into 3 groups of 5–8 mice each: (I) Ctrl (control), (II) CIA (collagen-induced arthritis model), and (III) CIA + Res (CIA and resveratrol-treated model). HE staining of mouse joints. **B** HE staining of mouse lungs. **C** Masson staining of mouse lung. **D** Mouse lung tissue qRT-PCR assay **E** Mouse lung tissue hydroxyproline assay. **F** Immunohistochemical detection of CollagenI and TGF-β1 protein expression in mouse lung tissue. **G** Western blot detection of CollagenI protein expression in mouse lung tissue. **H** Western blot for TGF-β1 protein expression in mouse lung tissue. *P < 0.05; **P < 0.01; ***P < 0.001

Earlier studies have reported the effectiveness of resveratrol in mitigating disease progression in rheumatoid arthritis [10]. Nevertheless, the potential effect of resveratrol in RA-associated interstitial lung disease (RA-ILD) is seldom addressed. In this study, we established a mouse model of RA-ILD treated with resveratrol. Hematoxylin and eosin (HE) staining of mouse joints revealed that resveratrol mitigated inflammation and damage in these joints (Fig. 1A). Both HE staining and Masson staining of lung tissue demonstrated the effective attenuation of fibrosis in RA-ILD by resveratrol (Fig. 1B, C). qRT-PCR results indicated a significant inhibition by resveratrol in the mRNA expression of Collagen I, Collagen III, and FN1 (Fig. 1D). Hydroxyproline assay further demonstrated the attenuation of fibrosis in RA-ILD by resveratrol (Fig. 1E). Additionally, immunohistochemistry and Western blot results illustrated that resveratrol not only decreased Collagen I expression but also markedly inhibited TGF-β1 expression (Fig. 1F–H).

### Resveratrol targeted inhibition of TGF-β1 alleviates RA-ILD progression

**Fig. 2:**
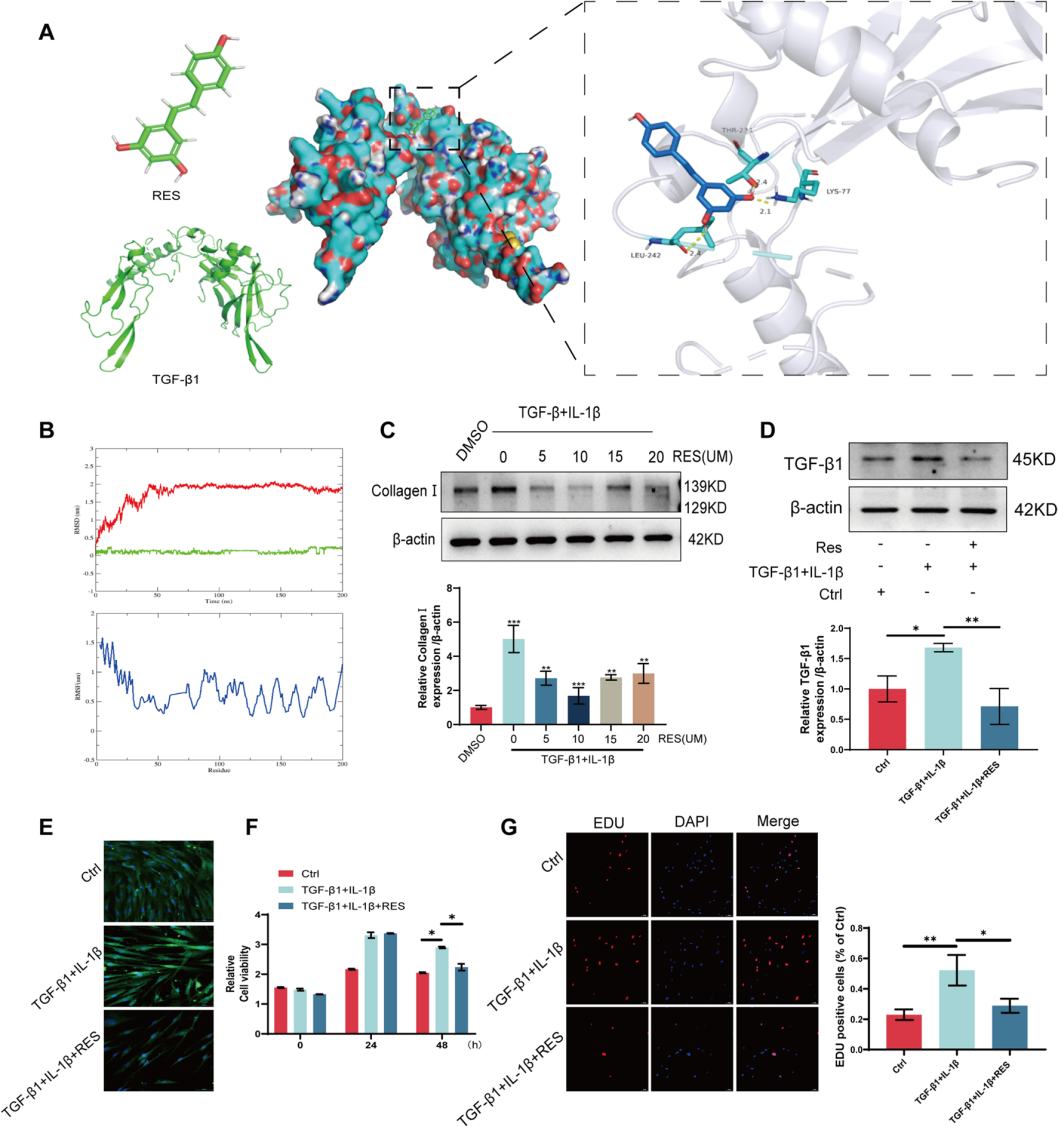
Resveratrol targets TGF-β1 to alleviate RA-ILD progression. **A** Molecular docking modeling of resveratrol with TGF-β1. **B** Molecular dynamics simulation verified the binding ability between resveratrol and TGF-β1 protein. **C** An in vitro cell model was constructed using TGF-β1 + IL-1β-induced MRC-5 cells, and Western blot detected Collagen I protein expression in the resveratrol concentration gradient-treated MRC-5 cell model. **D** Western blot detection of TGF-β1 protein expression in MRC-5 cell model after resveratrol treatment. **E** Immunofluorescence detection of TGF-β1 protein expression in MRC-5 cell model after resveratrol treatment. **F** CCK8 detection of viability of MRC-5 cell model after resveratrol treatment. **G** EDU detection of cell cycle in MRC-5 cell model after resveratrol treatment. *P < 0.05; **P < 0.01; ***P < 0.001

To assess the potential of resveratrol in mitigating the progression of RA-ILD through the inhibition of TGF-β1 production, we developed a molecular docking model of resveratrol targeting TGF-β1. The binding free energy of the model complex was − 5.8 kcal/mol, indicating a relatively stable binding affinity between resveratrol and TGF-β1 protein (Fig. 2A). Molecular dynamics simulation confirmed the stability of the binding between resveratrol and TGF-β1 protein. The equilibrium of the simulation system was evaluated using RMSD, revealing that the protein complex started to stabilize at 50 ns. RMSF analysis reflected the freedom degree of each residue within the molecule. These findings collectively suggest that resveratrol exerts its pharmacological effects through direct binding to the TGF-β1 protein (Fig. 2B). Subsequently, we induced MRC-5 cells with TGF-β1 + IL-1β (10 ng/mL + 5 ng/mL) to establish an in vitro model. Through Western blot and immunofluorescence experiments, we identified the optimal concentration of resveratrol (10 µm) and evaluated its effect on TGF-β1 protein expression in the cell model. The results indicated a significant inhibition by resveratrol in Collagen I and TGF-β1 protein expression in MRC-5 cells (Fig. 2C–E). Furthermore, CCK8 and EDU assays demonstrated that resveratrol attenuated TGF-β1-induced cell proliferation (Fig. 2F, G). The aforementioned findings suggest that resveratrol alleviates fibrosis in RA-ILD by targeting and inhibiting the expression of TGF-β1.

### Resveratrol attenuates inflammation and oxidative stress in RA-ILD

**Fig. 3:**
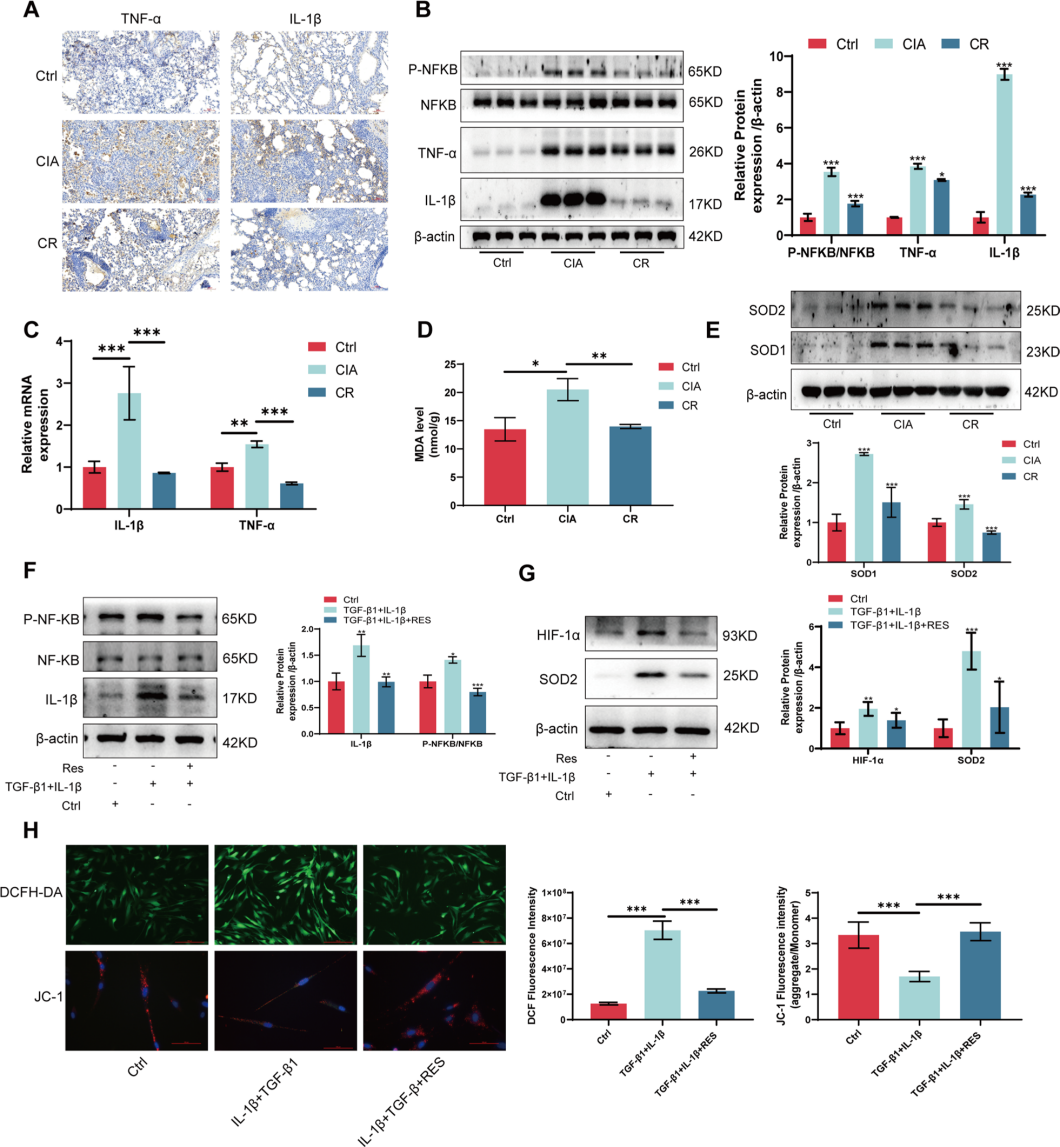
Resveratrol attenuates inflammation and oxidative stress in RA-ILD. **A** Immunohistochemical detection of TNF-α and IL-1β protein expression in lung tissue. **B** Western blotting was used to detect P-NFKB, NFKB, TNF-α, and IL-1β protein expression in lung tissue. **C** qRT-PCR for TNF-α, IL-1β RNA expression in lung tissue. **D** MDA detects lung tissue oxidation levels. **E** Western blotting to detect SOD1 and SOD2 protein expression in lung tissue. **F** Western blotting to detect P-NFKB, NFKB, IL-1β protein expression in MRC-5 cell model after resveratrol treatment. **G** Western blotting to detect HIF-1α, SOD2 protein expression in the MRC-5 cell model after resveratrol treatment. **H** DCFH-DA and JC-1 were used to detect the levels of ROS and membrane potential in the MRC-5 cell model after resveratrol treatment, respectively. *P < 0.05; **P < 0.01; ***P < 0.001

To assess whether resveratrol mitigated inflammation and oxidative stress in RA-ILD, we analyzed the expression of inflammation-related proteins (e.g., TNF-α and IL-1β) using immunohistochemistry, Western blotting, and qRT-PCR. The findings demonstrated that resveratrol reduced inflammation in RA-ILD (Fig. 3A–C). Furthermore, the results from MDA and Western blotting experiments confirmed the reduction in oxidative stress in RA-ILD due to resveratrol treatment (Fig. 3D, E). To further corroborate the alleviation of inflammation and oxidative stress by resveratrol in RA-ILD, we assessed the expression of inflammation and oxidative stress-related proteins in an in vitro cellular model. The results were consistent with the in vivo animal experiments, confirming that resveratrol reduced inflammation and oxidative stress in MRC-5 cells (Fig. 3F, G). Moreover, ROS and JC-1 assays revealed that resveratrol decreased the levels of ROS and prevented the decline in cell membrane potential in MRC-5 cells (Fig. 3H). In summary, these findings indicate that resveratrol alleviated inflammation and oxidative stress in RA-ILD.

### Resveratrol restores autophagy abnormalities in RA-ILD

**Fig. 4:**
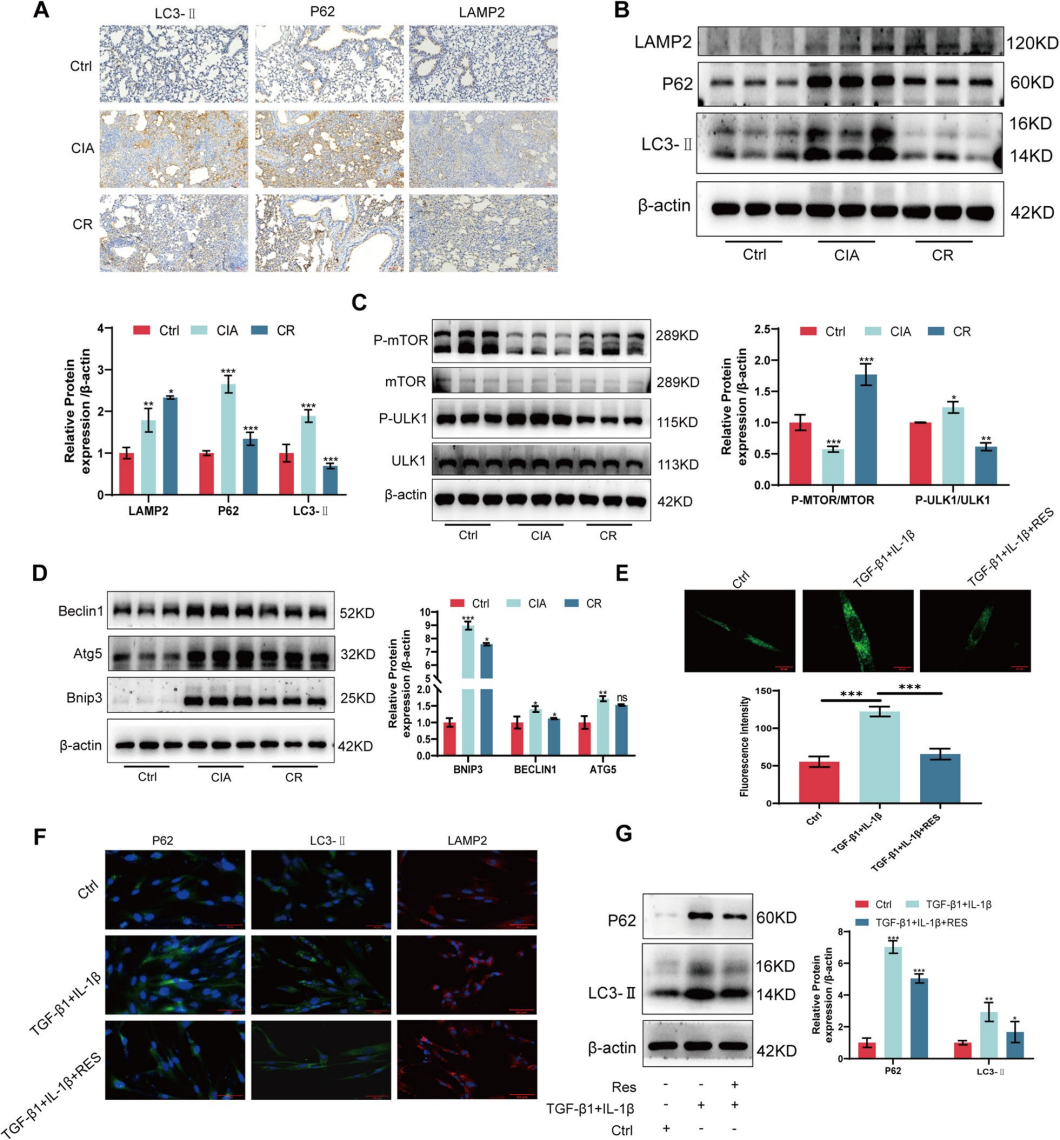
Resveratrol restores autophagy abnormalities in RA-ILD. **A** Immunohistochemical detection of LC3-II, P62, and LAMP2 protein expression in lung tissue. **B** Western blotting for LC3-II, P62, and LAMP2 protein expression in lung tissue. **C** Western blotting to detect P-mTOR, mTOR, P-ULK1, and ULK1 protein expression in lung tissues. **D** Western blotting to detect Beclin1, Atg5, and Bnip3 protein expression in lung tissue. **E** MDC detection of autophagic vesicle changes in the MRC-5 cell model after resveratrol treatment. **F** Immunofluorescence detection of LC3-II, P62, and LAMP2 protein expression in MRC-5 cell model after resveratrol treatment. **G** Western blot detection of LC3-II, P62 protein expression in MRC-5 cell model after resveratrol treatment. *P < 0.05; **P < 0.01; ***P < 0.001

The study revealed a notable alteration in autophagy levels in RA-ILD, as depicted in the figure below. Immunohistochemistry and Western blotting demonstrated substantial accumulation of P62 in RA-ILD, along with increased expression of LC3-II and LAMP2. However, resveratrol treatment resulted in reduced levels of P62 and LC3-II, with a slight increase in LAMP2 expression (Fig. 4A, B). Furthermore, resveratrol facilitated mTOR activation, inhibited ULK1 phosphorylation (Fig. 4C), and reinstated the expression of Beclin1, Atg5, and Bnip3, which exhibited elevated levels in RA-ILD (Fig. 4D). To further confirm the modulation of autophagy in RA-ILD by resveratrol, we assessed the presence of autophagic vesicles in cell models using MDC (Fig. 4E) and analyzed the protein expression of P62, LC3-II, and LAMP2 through Western blotting and immunofluorescence (Fig. 4F, G). The findings indicated that resveratrol reinstated the disrupted autophagic flux in RA-ILD.

### Resveratrol attenuates RA-ILD by restoring autophagic flux through promoting autophagic lysosomal fusion

**Fig. 5:**
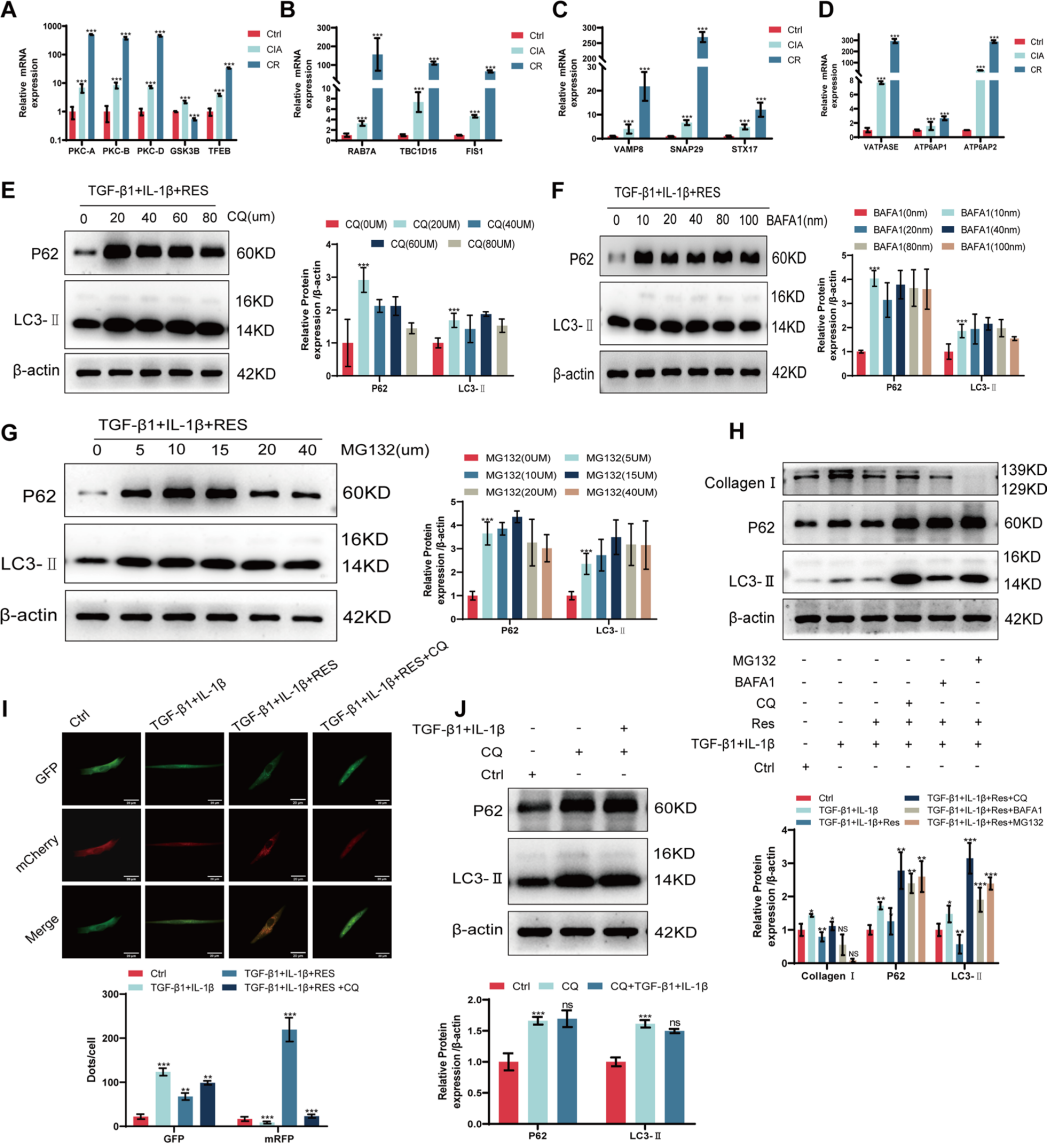
Resveratrol attenuates RA-ILD by promoting autophagic lysosomal fusion to restore autophagic flux. **A** qRT-PCR for RNA expression of PKC-A, PKC-B, PKC-D, GSK3B and TFEB in lung tissue. **B** qRT-PCR for lung tissue RAB7A, TBC1D15, FIS1 RNA expression. **C** qRT-PCR to detect lung tissue VAMP8, SNAP29, STX17 RNA expression. **D** qRT-PCR to detect VATPASE, ATP6AP1, ATP6AP2 RNA expression in lung tissues. **E** Western blotting to detect P62, LC3-II protein expression in MRC-5 cells after CQ concentration gradient treatment with resveratrol. **F** Western blotting detection of P62, LC3-II protein expression in MRC-5 cells after BAFA1 concentration gradient treatment with resveratrol. **G** Western blotting detection of P62, LC3-II protein expression in MRC-5 cells after resveratrol treatment with MG132 concentration gradient. **H** Western blotting detection of P62, LC3-II, Collagen I protein expression in MRC-5 cells after resveratrol treatment with CQ, BAFA1, MG132. **I** mCherry-GFP-LC3 detection in MRC-5 cells after CQ treatment with resveratrol. **J** Western blot detection of P62, LC3B protein expression in CQ + TGF-β1 + IL-1β-treated MRC-5 cells. *P < 0.05; **P < 0.01; ***P < 0.001

To further elucidate the role of resveratrol in autophagy in RA-ILD, we assessed the expression of autophagy-related RNAs, including TFEB, RAB7A, VAMP8, and VATPASE, using qRT-PCR. The results revealed elevated expression of pro-autophagy lysosomal fusion RNAs in RA-ILD, which was further augmented upon resveratrol treatment (Fig. 5A–D). Subsequently, MRC-5 cells were treated with chloroquine (CQ), bafilomycin A1 (BAFA1), and MG132, and we determined the optimal concentrations (CQ: 20 µm, BAFA1: 40 nm, MG132: 10 µm) based on autophagy protein levels (Fig. 5E–G). We assessed the impact of these three drugs on the expression of autophagy proteins and Collagen I in the cellular model following resveratrol treatment using Western blotting. The results indicated that CQ significantly impeded the effects of resveratrol when compared to the other two drugs, resulting in autophagy and fibrosis levels akin to those observed in RA-ILD (Fig. 5H). To further confirm these findings, we transfected the mCherry-GFP-LC3 plasmid into MRC-5 cells and assessed the progress of autophagy and lysosomal fusion based on alterations in fluorescence (Fig. 5I). In addition, to determine whether the apparent upregulation of LC3-II was due to inhibition of autophagic flux rather than initiation of normal autophagy. We performed experiments using CQ plus TGF-β1 + IL-1β, and the results showed that compared to the CQ group, the LC3-II protein in the CQ + TGF-β1 + IL-1β group was not significantly increased or even slightly decreased, and the P62 protein was not significantly altered. Thus indicating that the upregulation of LC3-II was not due to TGF-β1 + IL-1β initiating normal cellular autophagy (Fig. 5J).

### TMEM175 promotes RA-ILD progression by inhibiting autophagic lysosomal fusion

**Fig. 6:**
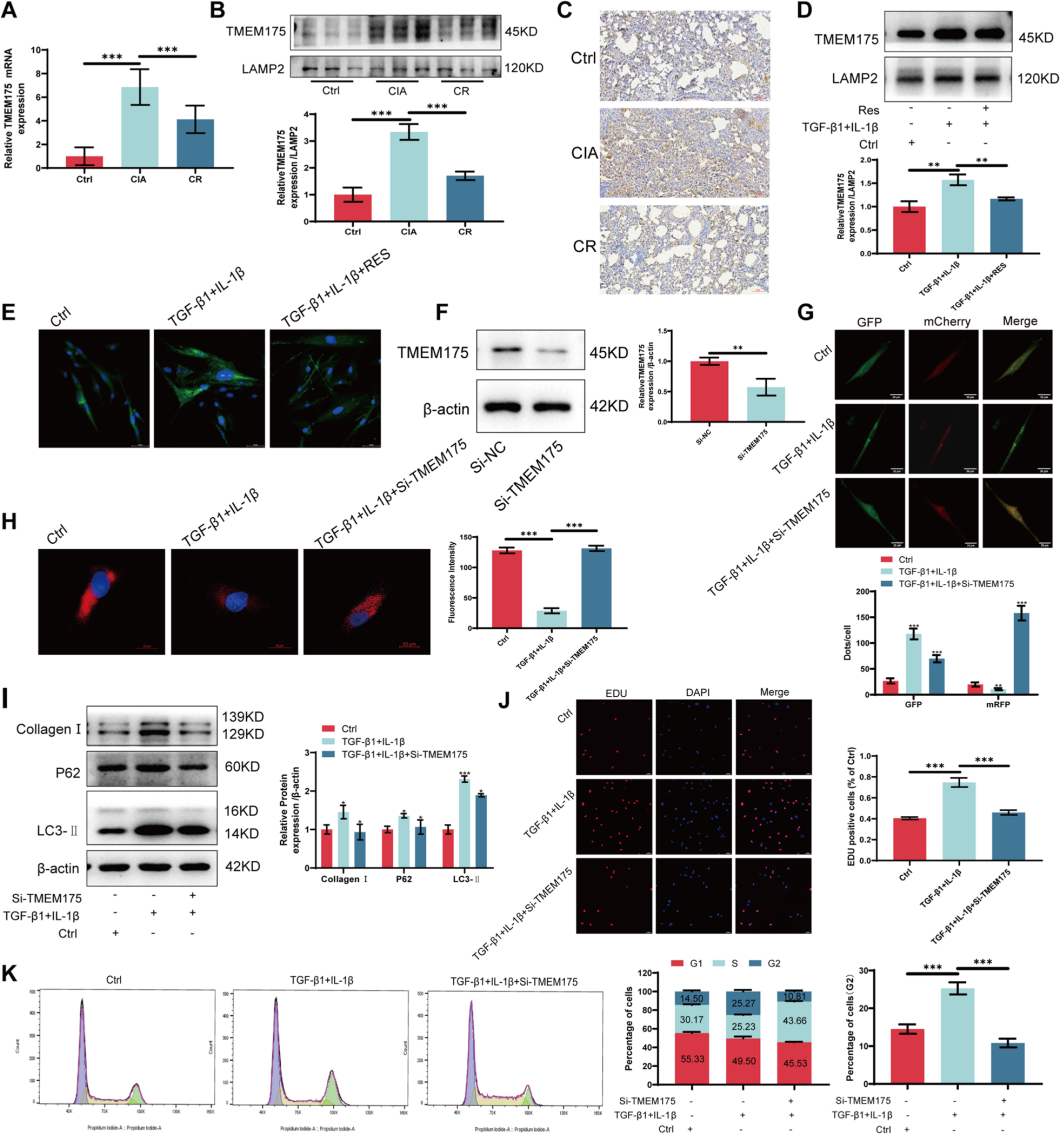
TMEM175 promotes RA-ILD progression by inhibiting autophagic lysosomal fusion. **A** qRT-PCR to detect TMEM175 RNA expression in lung tissues. **B** Western blotting to detect TMEM175 protein expression in lung tissues. **C** Immunohistochemistry for TMEM175 protein expression in lung tissue. **D** Western blotting to detect TMEM175 protein expression in MRC-5 cells after resveratrol treatment. **E** Immunofluorescence to measure TMEM175 protein expression in MRC-5 cells after resveratrol treatment. **F** Western blotting to detect TMEM175 protein expression after interference. **G** mCherry-GFP-LC3 detection of autophagy changes after interference with TMEM175. **H** Lyso-Tracker Red staining to detect lysosomal changes after TMEM175 interference. **I** Western blotting detection of LC3-II, P62, and Collagen I protein expression after interference with TMEM175. **J** EDU detection of cell proliferation after interference with TMEM175. **K** Flow cytometry detection of cell cycle after interference with TMEM175. *P < 0.05; **P < 0.01; ***P < 0.001

To further investigate how resveratrol regulates the fusion of autophagic lysosomes, we found that TMEM175 was highly expressed in RA-ILD using qRT-PCR and showed a decrease in the resveratrol-treated group (Fig. 6A). Western blotting with immunohistochemistry confirmed this result (Fig. 6B, C). Next, we re-evaluated TMEM175 expression in an in vitro cellular model, which showed consistent results with the previous findings (Fig. 6D, E). To delve deeper into the mechanism through which TMEM175 regulates autophagic lysosomal fusion, we employed SiRNA interference. Western blotting was employed to assess the impact of interference (Fig. 6F). Analysis of cellular autophagy following interference with TMEM175 using mCherry-GFP-LC3 labeling demonstrated the restoration of autophagic lysosomal fusion in MRC-5 cells (Fig. 6G). Furthermore, Lyso-Tracker Red staining revealed a decrease in acidic lysosomes within the TGF-β1-induced MRC-5 cell model, and this effect was counteracted by TMEM175 interference (Fig. 6H). Conversely, Western blotting results demonstrated that interference with TMEM175 restored autophagy and suppressed fibrosis in MRC-5 cells (Fig. 6I). The outcomes of EDU staining and flow cytometry assays indicated that interference with TMEM175 suppressed TGF-β1-induced cell proliferation (Fig. 6J, K). These findings imply that resveratrol mitigates fibrosis in RA-ILD by suppressing TMEM175 protein expression, restoring lysosomal pH, and enhancing autophagic lysosomal fusion.

### AKT assists TMEM175 in the regulation of autophagic lysosomal fusion

**Fig. 7:**
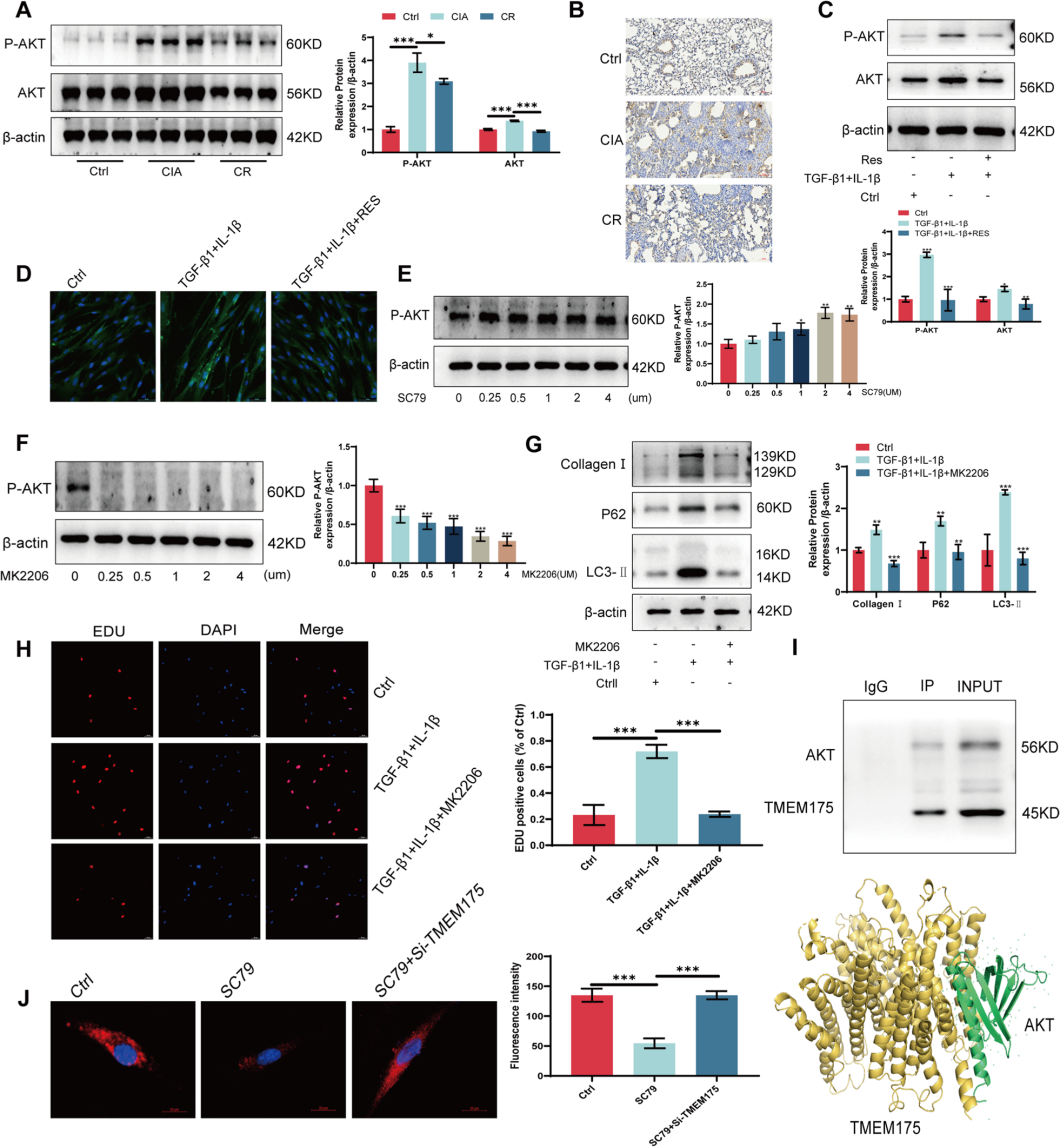
AKT assists TMEM175 in the regulation of autophagic lysosomal fusion. **A** Western blotting to detect P-AKT and AKT protein expression in lung tissue. **B** Immunohistochemistry to detect AKT protein expression in lung tissue. **C** Western blotting to detect P-AKT, AKT protein expression in MRC-5 cells after resveratrol treatment. **D** Immunofluorescence detection of AKT protein expression in MRC-5 cells after resveratrol treatment. **E** Western blotting to detect P-AKT protein expression in SC79 concentration gradient-treated MRC-5 cells. **F** Western blotting detection of P-AKT protein expression in MRC-5 cells treated with MK2206 concentration gradient. **G** Western blotting detection of LC3-II, P62, Collegen I protein expression in MRC-5 cells treated with MK2206. **H** EDU detection of cell proliferation after MK2206 treatment of MRC-5 cells. **I** CO-IP detection of AKT binding to TMEM175 protein. **J** Lyso-Tracker Red staining detects lysosomal changes after co-treatment of SC79 with interfering TMEM175 in MRC-5 cells. *P < 0.05; **P < 0.01; ***P < 0.001

Prior studies have reported the formation of a lysosomal K^+^ channel complex when AKT binds with TMEM175. This complex is activated by growth factors and plays a role in regulating lysosomal K^+^ and H^+^ transport. This regulation occurs via an adaptable effector gate that influences lysosomal pH, thereby impacting autophagic lysosomal fusion [27, 28, 31, 32]. Consequently, we conducted immunohistochemistry and Western blotting on animal models to assess AKT expression levels. The results indicated elevated levels of both P-AKT and AKT in RA-ILD, which decreased following resveratrol treatment (Fig. 7A, B). We subsequently validated these findings through in vitro experiments, wherein we observed elevated AKT expression in TGF-β1-induced MRC-5 cells. Resveratrol treatment effectively reversed the AKT elevation (Fig. 7C, D). To further explore the role of AKT in RA-ILD, we treated MRC-5 cells with an AKT activator (SC79) and an AKT-specific inhibitor (MK2206). We determined the optimal drug concentrations (SC79: 2 μM, MK2206: 4 μM) through Western blotting analysis (Fig. 7E, F). Western blotting results indicated that inhibiting AKT metabolic activation reinstated TGFβ1-induced autophagy and decreased Collagen I expression in MRC-5 cells (Fig. 7G). Additionally, EDU assays demonstrated that inhibiting AKT metabolic activation reversed the proliferation of MRC-5 cells (Fig. 7H). Subsequently, we conducted co-immunoprecipitation experiments to illustrate the formation of a complex between AKT and TMEM175. This complex plays a role in regulating autophagic lysosomal fusion (Fig. 7I). Additionally, Lyso-Tracker Red staining revealed that AKT activation facilitated the formation of the AKT/MEM175 complex and led to a reduction in the number of acidic lysosomes. However, this effect was reversed upon disruption of TMEM175 (Fig. 7J). To summarize, AKT serves as a gating control, facilitating the opening of the TMEM175 ion channel, and consequently, it regulates lysosomal H^+^ transport. However, it alone cannot independently influence lysosomal pH changes.

## Discussion

In this study, we investigated the connection between RA-ILD and autophagy, as well as the underlying mechanism through which resveratrol reinstates autophagic flux via the AKT/TMEM175 pathway. Our findings demonstrated that resveratrol decreased the activation of the AKT/MEM175 complex by suppressing AKT expression. This, in turn, restored autophagic lysosomal fusion, mitigated fibrosis in RA-ILD, and reduced inflammation and oxidative stress levels. In this study, we employed an arthritis model induced by the combination of chicken type II collagen and Fuchs’ complete adjuvant. The model group exhibited elevated levels of TGF-β1, AKT, TMEM175, fibrosis, inflammation, and oxidation. Additionally, there impeded autophagic flux, which was alleviated by resveratrol treatment. In our in vitro cellular model, we stimulated MRC-5 cells with TGF-β1 + IL-1β to replicate RA-ILD conditions. Our experimental results indicated that both TGF-β1 and IL-1β treatments upregulated AKT expression and activated the AKT/MEM175 ion channel. This activation led to an increase in lysosomal H^+^ efflux and a hindrance in autophagic lysosomal fusion, which was counteracted by TMEM175 interference, ultimately alleviating fibrosis progression. In summary, our findings suggest that resveratrol diminishes inflammation and fibrosis in rheumatoid arthritis-associated interstitial lung disease by reinstating autophagic lysosomal flux through the AKT/TMEM175 pathway.

RA-ILD is a pulmonary condition resulting from an inflammatory response, which can lead to fibrosis and scarring of the lung interstitium, ultimately causing impaired lung function [31–33]. Data indicates a decreasing overall mortality rate in RA, yet the mortality rate in RA-ILD seems to be steadily increasing over the years. The precise etiology of RA-ILD remains unknown; however, it is believed that the inflammatory response and abnormal immune system activation play significant roles in its pathogenesis [34, 35]. In our study, we demonstrated elevated protein levels of IL-1β, TNF-α, SOD1, and SOD2 in RA-ILD. Furthermore, we observed a significant impairment in autophagic flux, increased expression of LC3-II and LAMP2, and substantial P62 accumulation in RA-ILD. This accumulation appeared to hinder autophagic lysosomal fusion and impede collagen degradation. These findings indicate that cellular autophagy plays a crucial role in the development of RA-ILD.

Resveratrol occurs naturally in certain plants and is a polyphenolic phytonutrient [36, 37]. Resveratrol is recognized as a natural antioxidant that can combat damage caused by oxygen-free radicals, thereby reducing oxidative stress and inflammation [38, 39]. Several studies propose that resveratrol may offer cardiovascular health benefits by potentially reducing cholesterol and blood pressure levels and preventing cardiovascular diseases like atherosclerosis [40]. Resveratrol is also considered to possess anti-cancer potential, as it inhibits the proliferation and metastasis of cancer cells while promoting apoptosis [41, 42]. Furthermore, resveratrol has demonstrated effectiveness in treating fibrosis in interstitial lung disease, as it mitigates the condition by reducing inflammation and oxidative stress level [43, 44]. The aforementioned studies offer compelling evidence supporting the feasibility of using resveratrol in the treatment of RA-ILD. Our investigation revealed that resveratrol decreased the expression of proteins including IL-1β, TNF-α, SOD1, and SOD2. It also restored autophagic blockage and reduced the accumulation of P62 and Collagen I protein. These findings imply that resveratrol inhibited fibrosis, decreased inflammation, and alleviated oxidative stress in RA-ILD by reinstating autophagic flux.

Autophagy is a vital cellular process responsible for breaking down and eliminating waste and damaged cellular components, ensuring the stability of the cell’s internal environment. Recently, there has been increasing interest in researching the relationship between lung diseases and autophagy [45]. Autophagy plays a role in conferring resistance to alveolar macrophage apoptosis and is implicated in the advancement of pulmonary fibrosis [46]. Deficiency of PINK1 disrupts mitochondrial homeostasis and facilitates the development of lung fibrosis [47]. Diosgenin mitigates inflammation and fibrosis in silica-induced lung conditions by enhancing autophagy in alveolar macrophages [48]. In our current investigation, we observed a substantial accumulation of autophagic vesicles containing collagen. We also noted an increase in lysosomal expression, a reduction in acidic lysosomes in RA-ILD, and a blockade in autophagic lysosomal fusion. However, treatment with resveratrol restored autophagic lysosomal fusion, enhanced autophagic flux, and alleviated fibrosis in RA-ILD. These findings indicate that resveratrol plays a role in modulating autophagy and is implicated in the progression of RA-ILD.

TMEM175 (Transmembrane Protein 175) is a transmembrane protein located in lysosomal membranes, serving as an ion channel crucial for preserving lysosomal acidity. It has been demonstrated to collaborate with the V-ATPase enzyme in the regulation of luminal pH within lysosomes [49]. Multiple studies indicate a potential link between TMEM175 and the onset of Parkinson’s disease [27, 50]. Deficiency of TMEM175 disrupts both lysosomal and mitochondrial function and promotes the aggregation of alpha-synuclein [51]. Moreover, TMEM175 may play a role in the progression of neurodegenerative diseases [52]. These studies indicate that TMEM175 may play a role in disease regulation by influencing lysosomal function. In our current study, we observed up-regulation of AKT, TMEM175, and TGF-β1 in RA-ILD, which was accompanied by a reduction in autophagic lysosomal fusion and an increase in fibrosis. However, these effects were reversed with resveratrol treatment. We also discovered that TMEM175 can form a complex with AKT and is activated by TGF-β1, which is involved in regulating lysosomal pH. These findings suggest that resveratrol diminishes the activation of the AKT/MEM175 complex, maintains lysosomal pH stability, restores autophagic flux, and mitigates fibrosis in RA-ILD by inhibiting the expression of TGF-β1.

Nonetheless, our current study does have certain limitations. Firstly, our focus was primarily on investigating the impact of resveratrol on autophagy and fibrosis in RA-ILD, while the underlying mechanisms through which resveratrol mitigates inflammation and oxidative stress in RA-ILD have not been thoroughly explored. Secondly, our research concentrated solely on the effects of resveratrol on human embryonic lung cells, without assessing its influence on immune cells like alveolar macrophages. Thirdly, more comprehensive investigations are warranted to delve into the regulatory mechanisms by which resveratrol reinstates autophagic lysosomal fusion.

## Conclusion

Our study offers the potential of resveratrol as an effective therapeutic agent for RA-ILD. Our results indicate that resveratrol plays a role in the regulation of fibrosis progression in RA-ILD by restoring autophagic lysosomal fusion. Moreover, we have elucidated the intrinsic mechanism by which resveratrol restores autophagic lysosomal flux to alleviate RA-ILD through the AKT/TMEM175 pathway. This clarification provides a theoretical foundation for the identification of more precise therapeutic targets for RA-ILD.

## Chemical compounds studied in this article

Resveratrol (PubChem CID: 445154), chloroquine (PubChem CID: 2719), bafilomycin A1 (PubChem CID: 6436223), MG132 (PubChem CID: 462382), SC79 (PubChem CID: 2810830), MK2206 (PubChem CID: 24964624).

## Data Availability

Data will be made available on request.

## Abbreviations

RA-ILD: Interstitial lung disease associated with rheumatoid arthritis
RA: Rheumatoid arthritis
ILD: Interstitial lung disease
Res: Resveratrol
TGF-β1: Transforming growth factor beta 1
TMEM175: Transmembrane protein 175
AKT: Protein kinase B
CFA: Complete Freund’s adjuvant
CCII: Chicken collagen II
H&E: Hematoxylin–eosin
ROS: Reactive oxygen species
Ctrl: Control
CIA: Collagen-induced arthritis models
CIA + Res: CIA and resveratrol-treated models
SiRNA: Small interfering RNA
Si-TMEM175: Interfering RNA of TMEM175
CQ: Chloroquine
BAFA1: Bafilomycin A1
BSA: Bovine serum albumin
SD: Standard deviation

## References

[1] Scherer HU, Häupl T, Burmester GR (2020). The etiology of rheumatoid arthritis. J Autoimmun.

[2] Gravallese EM, Firestein GS (2023). Rheumatoid arthritis—common origins, divergent mechanisms. N Engl J Med.

[3] Graudal N, Nielsen CT, Lindhardsen J (2023). Pirfenidone in rheumatoid arthritis-associated interstitial lung disease. Lancet Respir Med.

[4] McDermott GC, Doyle TJ, Sparks JA (2021). Interstitial lung disease throughout the rheumatoid arthritis disease course. Curr Opin Rheumatol.

[5] Kadura S, Raghu G (2021). Rheumatoid arthritis-interstitial lung disease: manifestations and current concepts in pathogenesis and management. Eur Respir Rev.

[6] Luppi F (2022). Acute exacerbation of interstitial lung disease associated with rheumatic disease. Nat Rev Rheumatol.

[7] Behl T (2022). Exploring the role of polyphenols in rheumatoid arthritis. Crit Rev Food Sci Nutr.

[8] Pastor RF (2019). Resveratrol, human health and winemaking perspectives. Crit Rev Food Sci Nutr.

[9] Riveiro-Naveira RR (2016). Resveratrol lowers synovial hyperplasia, inflammatory markers and oxidative damage in an acute antigen-induced arthritis model. Rheumatology.

[10] Yang G (2018). Resveratrol alleviates rheumatoid arthritis via reducing ROS and inflammation, inhibiting MAPK signaling pathways, and suppressing angiogenesis. J Agric Food Chem.

[11] Wang G (2020). Resveratrol ameliorates rheumatoid arthritis via activation of SIRT1-Nrf2 signaling pathway. BioFactors.

[12] Levine B, Kroemer G (2019). Biological functions of autophagy genes: a disease perspective. Cell.

[13] Li W (2021). Selective autophagy of intracellular organelles: recent research advances. Theranostics.

[14] Li B (2023). Ultrasound-remote selected activation mitophagy for precise treatment of rheumatoid arthritis by two-dimensional piezoelectric nanosheets. ACS Nano.

[15] Keller CW, Adamopoulos IE, Lünemann JD (2023). Autophagy pathways in autoimmune diseases. J Autoimmun.

[16] Manganelli V (2018). Autophagy induces protein carbamylation in fibroblast-like synoviocytes from patients with rheumatoid arthritis. Rheumatology.

[17] Lee WS (2020). Protective role of optineurin against joint destruction in rheumatoid arthritis synovial fibroblasts. Arthritis Rheumatol.

[18] Guan R (2022). Bone morphogenetic protein 4 inhibits pulmonary fibrosis by modulating cellular senescence and mitophagy in lung fibroblasts. Eur Respir J.

[19] Baek AR (2020). Spermidine attenuates bleomycin-induced lung fibrosis by inducing autophagy and inhibiting endoplasmic reticulum stress (ERS)-induced cell death in mice. Exp Mol Med.

[20] Zhang J (2022). ATF3-activated accelerating effect of LINC00941/lncIAPF on fibroblast-to-myofibroblast differentiation by blocking autophagy depending on ELAVL1/HuR in pulmonary fibrosis. Autophagy.

[21] Bao L (2022). Resveratrol ameliorates fibrosis in rheumatoid arthritis-associated interstitial lung disease via the autophagy-lysosome pathway. Molecules.

[22] Cang C (2015). TMEM175 is an organelle K(+) channel regulating lysosomal function. Cell.

[23] Zhang J (2023). Lysosomal LAMP proteins regulate lysosomal pH by direct inhibition of the TMEM175 channel. Mol Cell.

[24] Perdigoto CN (2022). Parkinson’s disease risk protein TMEM175 keeps lysosomes running on a proton leak. Nat Struct Mol Biol.

[25] Hua H (2021). Targeting Akt in cancer for precision therapy. J Hematol Oncol.

[26] Zhao Z (2022). TGF-β promotes pericyte-myofibroblast transition in subretinal fibrosis through the Smad2/3 and Akt/mTOR pathways. Exp Mol Med.

[27] Wie J (2021). A growth-factor-activated lysosomal K(+) channel regulates Parkinson’s pathology. Nature.

[28] Hu M (2023). The acid gate in the lysosome. Autophagy.

[29] Ullrich F (2021). Growth factors AKTivate lysosomal TMEM175 K(+) channels. Nat Struct Mol Biol.

[30] Albanawany NM (2022). Histopathological, physiological and biochemical assessment of resveratrol nanocapsules efficacy in bleomycin-induced acute and chronic lung injury in rats. Drug Deliv.

[31] Spagnolo P (2021). Mechanisms of progressive fibrosis in connective tissue disease (CTD)-associated interstitial lung diseases (ILDs). Ann Rheum Dis.

[32] Oliveira RP (2022). Connective tissue disease-associated interstitial lung disease. Pulmonology.

[33] Doyle TJ (2015). Detection of rheumatoid arthritis-interstitial lung disease is enhanced by serum biomarkers. Am J Respir Crit Care Med.

[34] Spagnolo P (2018). The lung in rheumatoid arthritis: focus on interstitial lung disease. Arthritis Rheumatol.

[35] Wu X (2022). Serum proteomic profiling of rheumatoid arthritis-interstitial lung disease with a comparison to idiopathic pulmonary fibrosis. Thorax.

[36] Luca SV (2020). Bioactivity of dietary polyphenols: the role of metabolites. Crit Rev Food Sci Nutr.

[37] Fiod Riccio BV (2020). Characteristics, biological properties and analytical methods of trans-resveratrol: a review. Crit Rev Anal Chem.

[38] Chen Y (2021). Resveratrol and its derivative pterostilbene attenuate oxidative stress-induced intestinal injury by improving mitochondrial redox homeostasis and function via SIRT1 signaling. Free Radic Biol Med.

[39] Liu Y (2020). Resveratrol-loaded biopolymer core-shell nanoparticles: bioavailability and anti-inflammatory effects. Food Funct.

[40] Shah A (2016). Effect of resveratrol on metabolic and cardiovascular function in male and female adult offspring exposed to prenatal hypoxia and a high-fat diet. J Physiol.

[41] Zheng Y (2022). Curcumin- and resveratrol-co-loaded nanoparticles in synergistic treatment of hepatocellular carcinoma. J Nanobiotechnol.

[42] Li S (2023). Polyphenols as potential metabolism mechanisms regulators in liver protection and liver cancer prevention. Cell Prolif.

[43] Chen J (2016). Therapeutic effects of resveratrol in a mouse model of LPS and cigarette smoke-induced COPD. Inflammation.

[44] Liu L (2023). Effects of resveratrol on pulmonary fibrosis via TGF-β/Smad/ERK signaling pathway. Am J Chin Med.

[45] Ryter SW, Choi AM (2015). Autophagy in lung disease pathogenesis and therapeutics. Redox Biol.

[46] Larson-Casey JL (2016). Macrophage Akt1 kinase-mediated mitophagy modulates apoptosis resistance and pulmonary fibrosis. Immunity.

[47] Bueno M (2015). PINK1 deficiency impairs mitochondrial homeostasis and promotes lung fibrosis. J Clin Invest.

[48] Du S (2019). Dioscin alleviates crystalline silica-induced pulmonary inflammation and fibrosis through promoting alveolar macrophage autophagy. Theranostics.

[49] Wang Y, Li W, Wang Q (2023). Lysosome pH-regulated H(+) /K(+) switching channel involved in maintaining lysosomal homeostasis. Febs j.

[50] Lill CM (2015). Impact of Parkinson’s disease risk loci on age at onset. Mov Disord.

[51] Jinn S (2017). TMEM175 deficiency impairs lysosomal and mitochondrial function and increases α-synuclein aggregation. Proc Natl Acad Sci USA.

[52] Wu L (2023). TMEM175: a lysosomal ion channel associated with neurological diseases. Neurobiol Dis.

